# Pregnancy does not adversely impact diagnostic tests for HTLV-1/2 infection

**DOI:** 10.1371/journal.pntd.0007736

**Published:** 2019-09-12

**Authors:** Carolina Rosadas, Jennifer H. Tosswill, Richard Tedder, Graham P. Taylor

**Affiliations:** 1 Department of Medicine, Imperial College London, London, United Kingdom; 2 Virus Reference Department, Public Health England, London, United Kingdom; Hospital Universitário Professor Edgard Santos, BRAZIL

## Abstract

Mother-to-child-transmission (MTCT) of human T-cell lymphotropic virus type-1(HTLV-1) contributes disproportionately to the burden of HTLV-1 associated diseases. All preventive measures to avoid MTCT rely on the identification of infected mothers. However, the impact of pregnancy on HTLV-1 diagnosis has not been clearly assessed. Paired samples from 21 HTLV-1 infected women taken during pregnancy and while not pregnant were analysed by CMIA and PCR. The signal-to-cut-off values (S/CO) were higher during pregnancy than in the paired non-pregnant samples. HTLV-1 proviral load did not alter significantly by pregnant state. S/CO positively correlated with HTLV proviral load. Pregnancy does not impair the diagnosis of HTLV-1/2, by either immunological (CMIA) or molecular (qPCR/nPCR) tests.

## Author summary

Human T-cell lymphotropic virus (HTLV) can be transmitted from mother to child, mainly by breastfeeding. All preventive measures to avoid HTLV mother to child transmission depend on the identification of infected pregnant women. HTLV diagnosis is based on serological screening tests, confirmed by serology and/or molecular assays. The aim of the study was to verify whether pregnancy adversely impacts the identification of HTLV infection. Using paired samples from 21 women living with HTLV-1/2 obtained during pregnancy and while not pregnant we demonstrate that both serology and molecular assays perform equally well in both settings and can be use for the diagnosis of HTLV infection during pregnancy.

## Introduction

At least 5–10 million individuals are living with human T-cell lymphotropic virus type 1 (HTLV-1) in the world [1]. This virus can be transmitted through unprotected sexual intercourse, by exposure to infected lymphocytes in blood or tissue and by mother-to-child transmission (MTCT), mainly by breastfeeding. The latter, responsible for maintaining infection in successive generations, is associated with a disproportionately high risk of adult T cell leukaemia/lymphoma (ATL) [2]. Infection in early life is also linked to infective dermatitis in children as well as juvenile and adult cases of disabling HTLV-1-associated myelopathy/tropical spastic paraparesis (HAM/TSP). Treatment for these high morbidity diseases remains limited and once infection has occurred disease cannot be prevented. Thus, avoidance of transmission is essential. Blood donor screening has been implemented in many countries, however Japan alone has a national antenatal screening programme. Prevention of HTLV-1 MTCT relies on the identification of infected mothers prior to delivery. Diagnosis of HTLV-1/2 infection is based on screening (anti-HTLV antibody detection by enzyme linked immunoassay [ELISA] or chemiluminescent microparticle immunoassay [CMIA]) followed by confirmatory tests (HTLV gag and env antibodies by Western Blot [WB] and/or HTLV DNA amplification and detection by polymerase chain reaction [PCR])[3]. The current commercial assays cite high sensitivity and specificity. One reason given in the UK national screening committee’s recent decision not to implement HTLV-1 antenatal screening was a lack of data on the reliability of HTLV-1 diagnostics tests during pregnancy[4]. Conversely, studies of pregnant women are considered a more reliable indicator of HTLV-1 seroprevalence in the general population with 45 such studies cited in the ECDC report on the geographical distribution of areas with a high prevalence of HTLV-1 infection [5], with data from Europe showing consistently higher seroprevalence in the antenatal population compared with blood donors [3,6]. To address whether pregnancy impacts on the serological and molecular diagnosis of HTLV-1 infection the results from testing samples taken from women when pregnant were compared with paired samples from the same women when not pregnant.

## Materials and methods

### Ethics statement

This study was conducted under the auspices of the communicable diseases research tissue bank and approved by the NRES Committee South Central–Oxford C (15/SC/0089). All participants signed a written informed consent.

### Methods

Twenty-one women attending the National Centre for Human Retrovirology, St. Mary’s Hospital, London, with either HTLV-1 or -2 infection donated blood samples. HTLV infection had in all cases been confirmed by Western Blot (Genelabs HTLV 2.4) in accordance with the manufacturer’s instructions, 19 had HTLV-1 and two HTLV-2 infection. Paired sera obtained during a pregnancy and whilst not pregnant were analysed according to the manufacturer’s instructions for anti-HTLV-1/2 antibodies detection using Abbott Architect rHTLV-I/II platform, a fully automated third generation CMIA that includes HTLV-1/2 recombinant proteins. The optical density (OD) of each sample compared to the negative/reactive cut-off value for each time point was recorded. HTLV-1 proviral load (PVL) in peripheral blood mononuclear cells (PBMCs) was determined by quantitative real time PCR (qPCR) targeting HTLV tax gene and human betaglobin gene as previously reported [7]. In those samples with an undetectable PVL by qPCR a nested PCR (nPCR) was used to detect and type HTLV DNA [8].

D'Agostino & Pearson omnibus normality test was used to verify whether the results had a Gaussian distribution. Paired t test and Wilcoxon matched pairs test were used to compare groups (pregnant and not pregnant) of parametric and non-parametric data, respectively. Spearman test was used to verify if there was a correlation between S/CO values and PVL.

### Results

**Fig 1:**
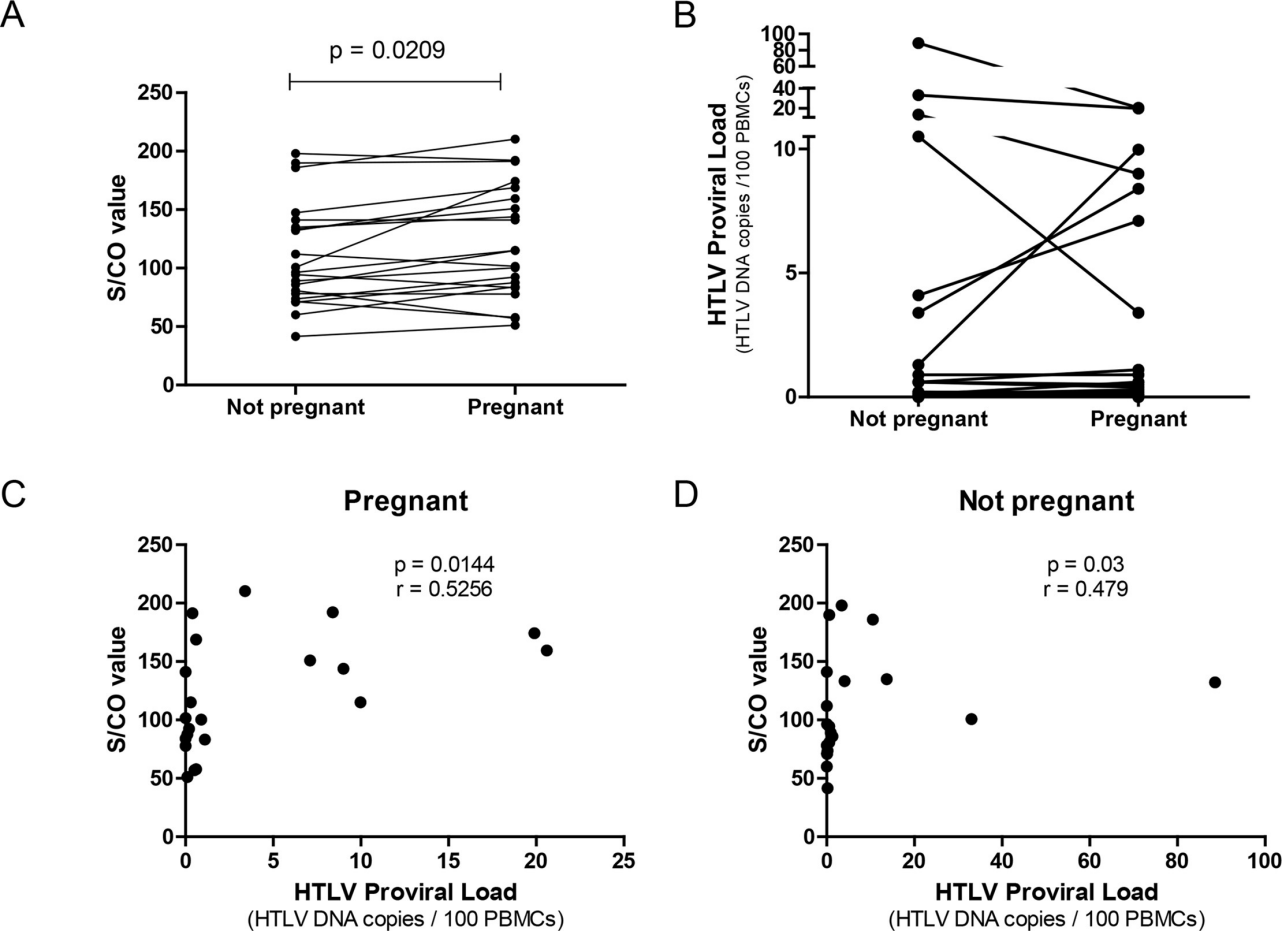
Comparative results of immunological and molecular tests of samples collected during pregnancy and while women were not pregnant. A) Sample/cut-off (S/CO) values (Architect rHTLV-I/II platform); B) HTLV proviral load (PVL) (qPCR); C) S/CO values and PVL correlation in pregnant women; D) S/CO values and PVL correlation in samples collected while women were not pregnant.

The clinical status, the trimester of testing and the serological and molecular results are presented in Table 1. Virtually all samples from non-pregnant time point were collected at least one year before delivery (varying from 1 year up to 10 years). Samples from patients number 1 and 9 were collected after delivery (7 days and 2.5 years after delivery, respectively) All samples were strongly positive by CMIA with a higher mean S/CO ratio 121.7 (SD 48.39) observed during pregnancy compared with the not pregnant time point 110.4 (SD 44.08); (p = 0.0209). The range of S/CO values was 51.1–210.3 during pregnancy and 41.46–198.1 in the samples collected when the women were not pregnant (Fig 1). The S/CO ratio was higher in 15/21 (71.4%) women during their pregnancy than in the paired non-pregnant sample. Third trimester serology did not differ significantly from the early trimesters.

**Table 1:** Summary of results of serological and molecular tests of samples obtained during pregnancy and while not pregnant.

ID	Clinical status	Trimester	S/CO pregnant	S/CO Not pregnant	Difference S/CO	PVL pregnant	PVL not pregnant	Difference PVL
1	AC	T3	100.4	89.1	11.3	0.9	0.9	0.0
2	AC	T2	115.0	96.4	18.6	0.3	0.1	0.2
3	AC	T3	150.9	133.2	17.7	7.1	4.1	3.0
4	AC	T3	191.3	189.9	1.3	0.4	0.6	-0.2
5	AC	T3	192.1	198.1	-5.9	8.4	3.4	5.0
6	AC	T3	83.2	94.4	-11.2	1.1	0.6	0.5
7	AC	T3	77.9	78.3	-0.4	0.0	0.0	0.0
8	AC	T1	92.4	73.7	18.7	0.2	0.2	0.0
9	AC	T3	57.8	71.3	-13.5	0.6	0.1	0.5
10	AC	T2	101.6	112.0	-10.4	0.0	0.0	0.0
11	AC	T2	57.0	80.7	-23.7	0.5	0.6	-0.1
12	AC	T2	168.8	147.4	21.4	0.6	NA	NA
13	AC	T3	141.24	141.20	0.04	0.0	0.0	0.0
14	AC	T3	143.9	134.8	9.1	9.0	13.7	-4.7
15	AC	T1	51.1	41.5	9.6	0.1	0.2	-0.1
16	AC	T2	115.0	85.9	29.1	10.0	1.3	8.7
17	HAM/TSP	T3	210.3	186.0	24.3	3.4	10.5	-7.1
18	HAM/TSP	T3	174.3	100.8	73.5	19.9	33.0	-13.1
19	ATL	T3	159.5	132.2	27.3	20.6	88.6	-68.0
20	HTLV-2	T2	87.7	71.0	16.7	0.1	0.1	0.0
21	HTLV-2	NA	84.1	60.1	24.0	0.0	0.0	0.0

S/CO: Signal-to-cut-off; PVL: Proviral load (HTLV-1 DNA copies per 100 PBMCs); AC: HTLV-1 asymptomatic carrier; HAM/TSP: patients with HTLV-1 associated myelopathy; ATL: patients with adult T cell leukaemia; HTLV-2: HTLV-2 infected individual, asymptomatic; NA: Not available

There was no significant difference in HTLV PVL in samples collected during pregnancy and while not pregnant (Median PVL (Interquartile range) HTLV-1 DNA copies per 100 PBMCs: Not pregnant 0.6 (3.85); pregnant: 0.6 (7.65); p = 0.8). The PVL was quantifiable in 17 women in whom it was lower during pregnancy in seven, the same in four and higher in six. Four women (3 HTLV-1 and 1 HTLV-2) did not have quantifiable HTLV PVL DNA in either sample of which: two women had HTLV-1 provirus DNA amplified and detected by nested PCR only in the sample from pregnancy; one did not have detectable HTLV-1 DNA in either sample, whilst in the patient with HTLV-2 proviral DNA was detected in 1/4 replicates, only in the sample obtained whilst pregnant.

HTLV PVL positively correlated with the antibody titre (S/CO) in both settings (Fig 1).

## Discussion

Pregnancy is a very specific physiological state in which the woman must tolerate the foetus by undergoing many immune and morphophysiological changes [9]. Immunoglobulin concentration in the serum decreases during pregnancy [10–12] and lower levels of antibody production are observed following influenza immunisation [13]. Haemodilution also results in physiological decrease in lymphocyte counts. Therefore, pregnancy might impact antibody production and could modulate the number of infected lymphocytes impacting HTLV proviral load. This could, in turn, hamper the diagnosis of pathogens by both immunological and molecular tests. Immunological assays to detect anti-HTLV-1 antibodies have been demonstrated by their respective manufacturers to have excellent sensitivity (usually considered 100%). The Abbott Architect rHTLV-I/II platform is a fully automated third generation assay with high sensitivity and specificity for HTLV-1/2 diagnosis in different clinical settings [14–16]. According to manufacturers, the interpretation of reactivity in Abbott Architect rHTLV-I/II is any signal-to-cut-off (S/CO) ratio of 1 or above. In the present study, this CMIA detected HTLV-1/2 antibodies in each of the women whilst pregnant. Furthermore, there was no evidence that anti-HTLV-1 antibody reactivity were lower in pregnancy. Indeed, in paired samples the S/CO values were overall higher during pregnancy. In an earlier study of 12,250 blood samples evaluated by CMIA a strong correlation with a S/CO above 20 and subsequent confirmation of HTLV-1/2 infection was observed, whereas no HTLV-1/2 infection was confirmed in patients where the S/CO was less than 4 [17]. The sera collected during pregnancy were not only reactive in the CMIA but all had high S/CO values with the lowest being 41.5. This points against a decrease in sensitivity for HTLV-1 diagnosis during pregnancy.

Once HTLV-1/2 reactivity has been detected the importance of further tests both to confirm and type HTLV-1/2 infection is well documented. Whilst more specific immunological tests such as WB and Immunofluorescence have been used as confirmatory tests for HTLV-1 infection (3) molecular tests are sometimes required especially for investigating indeterminate serology. In a recent study from Southern Brazil of 643 pregnant women Medeiros et al (18) reported that 0.6% tested positive in CMIA, of which 50% were confirmed by PCR. They found that negative-PCR samples had low S/CO values (1.3 and 2.5) in the Architect assay whereas the PCR-positive samples had high S/CO values (78.3 and 137.5) consistent with previously reported UK findings [18]. In our study, all sera had high S/CO values even those from the four patients with undetectable HTLV-1 and HTLV-2 DNA by qPCR. All patients had HTLV infection confirmed by WB and the one with a negative PCR was an asymptomatic carrier. From a diagnostic perspective it is important to realise that a proportion of carriers, HTLV elite controllers, have undetectable HTLV-1/2 DNA. However, from a transmission perspective we can consider these women to have low risk of HTLV-1 MTCT.

In a Japanese cohort HTLV-1 PVL was stable during pregnancy, with a slight increase after birth [19]. In our study, there was no statistically significant difference between samples collected during pregnancy and while not pregnant. The results in both CMIA and PCR were concordant in the paired samples. All four undetectable results by qPCR were undetectable in both settings. Thus, the risk of MTCT can be discussed prior to conception.

Strong correlation between antibody reactivity and PVL in blood was already described among pregnant women at delivery [20] and in non-pregnant HTLV-1 carriers [21]. The present study observes modest correlation in both settings. High antibody reactivity was assumed to be a risk factor for HTLV-1 MTCT. However, further studies showed that the association was due to the strong correlation among PVL and antibodies [20]. High PVL was reported as an independent risk factor associated with MTCT. The protective role of these antibodies (if any) and if they are transferred to the new-born need to be evaluated.

In conclusion, this study shows that pregnancy does not impair the diagnosis of HTLV-1/2, by either immunological (CMIA) or molecular (qPCR/nPCR) tests. Therefore, currently available tests can be used for antenatal screening and for confirmation of HTLV-1/2 infection allowing women to make informed decisions particularly in regard to infant feeding and onward transmission to their offspring.
